# Identification of novel tumor suppressor proteases by degradome profiling of colorectal carcinomas

**DOI:** 10.18632/oncotarget.1303

**Published:** 2013-09-15

**Authors:** Julia M. Fraile, Gonzalo R. Ordóñez, Pedro M. Quirós, Aurora Astudillo, José A. Galván, Dolors Colomer, Carlos López-Otín, José M.P. Freije, Xose S. Puente

**Affiliations:** ^1^ Departamento de Bioquímica y Biología Molecular, Facultad de Medicina, Instituto Universitario de Oncología, Universidad de Oviedo, 33006-Oviedo, Spain; ^2^ Servicio de Anatomía Patológica, Instituto Universitario de Oncología, Hospital Universitario Central de Asturias, Oviedo, Spain; ^3^ Banco de Tumores, Instituto Universitario de Oncología, Hospital Universitario Central de Asturias, Oviedo, Spain; ^4^ Hospital Clínic, IDIBAPS, University of Barcelona, Barcelona, Spain

**Keywords:** proteolysis, metastasis, invasion, cancer

## Abstract

Proteolytic enzymes play important roles during tumor development and progression through their ability to promote cell growth or by facilitating the invasion of surrounding tissues. The human genome contains more than 570 protease-coding genes, many of them forming functional networks, which has forced the use of global strategies for the analysis of this group of enzymes. In this study, we have designed a new quantitative PCR-based device for profiling the entire degradome in human malignancies. We have used this method to evaluate protease expression levels in colorectal carcinomas with the finding that most proteases with altered expression in these tumors exert their function in the extracellular compartment. In addition, we have found that among genes encoding repressed proteases there was a higher proportion with somatic mutations in colorectal cancer when compared to genes coding for upregulated proteases (14% vs. 4%, p<0.05). One of these genes, *MASP3*, is consistently repressed in colorectal carcinomas as well as in colorectal cancer cell lines when compared to normal colonic mucosa. Functional analysis of this gene revealed that ectopic expression of *MASP3* reduces cell proliferation *in vitro* and restrains subcutaneous tumor growth, whereas its downregulation induces an increase in the tumorigenic potential of colorectal cancer cells. These results provide new insights into the diversity of proteases associated with cancer and support the utility of degradome profiling to identify novel proteases with tumor-defying functions.

## INTRODUCTION

Proteases comprise a diverse group of enzymes with the ability to cleave peptide bonds. Their importance in human physiology is illustrated by their participation in numerous biological processes, including embryonic development and differentiation, cell proliferation and apoptosis, tissue remodelling and wound healing or cell migration and angiogenesis [1-2]. The analysis of different mammalian genomes has shown that protease-coding genes constitute about 2% of all protein-coding genes, with at least 570 proteases in human and other primates, and more than 630 in rodents [3-5]. This large number of genes, and the fact that many of them work together in specific networks, has led to the introduction of novel concepts for the global analysis of proteases. In this regard, the term degradome has been coined to define the complete set of protease genes present in a genome, and in the case of cancer, the tumor degradome constitutes the set of genes expressed by a tumor during disease progression [6-7].

Tumor proteases have been widely studied during the last decades due to their ability to cleave the extracellular matrix, facilitating tumor invasion and metastasis, or to activate specific cytokines and growth factors necessary for tumor growth and angiogenesis [8-10]. The relevance of proteases in cancer is underscored by the finding that a growing number of protease genes are mutated in different malignancies [11-17]. Furthermore, changes in the expression profile of proteolytic genes have been widely associated with tumor development. Thus, overexpression of *MMP1* or *MMP2* has been shown to be necessary to form lung metastasis by breast tumor cells [18] and expression of specific proteases is a hallmark of many tumor types [19-21]. Due to the initial discovery of proteases with tumor promoting activities, most expression profiling studies have focused their attention on proteases overexpressed by tumor cells, while little attention has been paid to proteases whose expression was repressed during malignant transformation. However, a growing body of evidence is showing that certain proteases can have tumor-defying functions, with some of them constituting *bona fide* tumor suppressors. This is the case of CYLD1, whose mutations cause cylindromatosis; A20, in which chromosomal deletions and inactivating mutations have been found in several lymphoma subtypes; BAP1, with point mutations and deletions described in breast and lung cancer and melanoma; CASP8, mutated in lymphoproliferative syndromes and different carcinomas, or USP7, implicated in p53 deubiquitylation [22-23]. Remarkably, some proteases hamper tumor growth or progression when either produced by tumor cells or by the tumor stroma [24-26]. In addition, the recent sequencing of cancer genomes is identifying novel somatic mutations affecting protease-coding genes [27-30], reinforcing the hypothesis that inactivation of certain proteases, by either somatic mutation or gene repression, might contribute to cancer development.

In this work, we have designed a new quantitative qPCR-based device for profiling the entire degradome in human. The use of a TaqMan-based approach allows a better quantification of differences in expression between biological samples, as well as provides an unmatched sensitivity to detect transcriptional changes affecting genes with low expression levels, which are usually difficult to determine when using traditional hybridization-based detection methods. We have used this new platform to assess and compare protease expression levels in normal mucosa and colorectal tumor samples. Thus, we have centered our attention on proteases whose expression was repressed in colorectal carcinoma providing the utility of degradome profiling as a good instrument to identify novel proteases with antitumor properties.

## RESULTS

### Expression of extracellular proteases is largely altered in colorectal carcinomas

**Figure 1:**
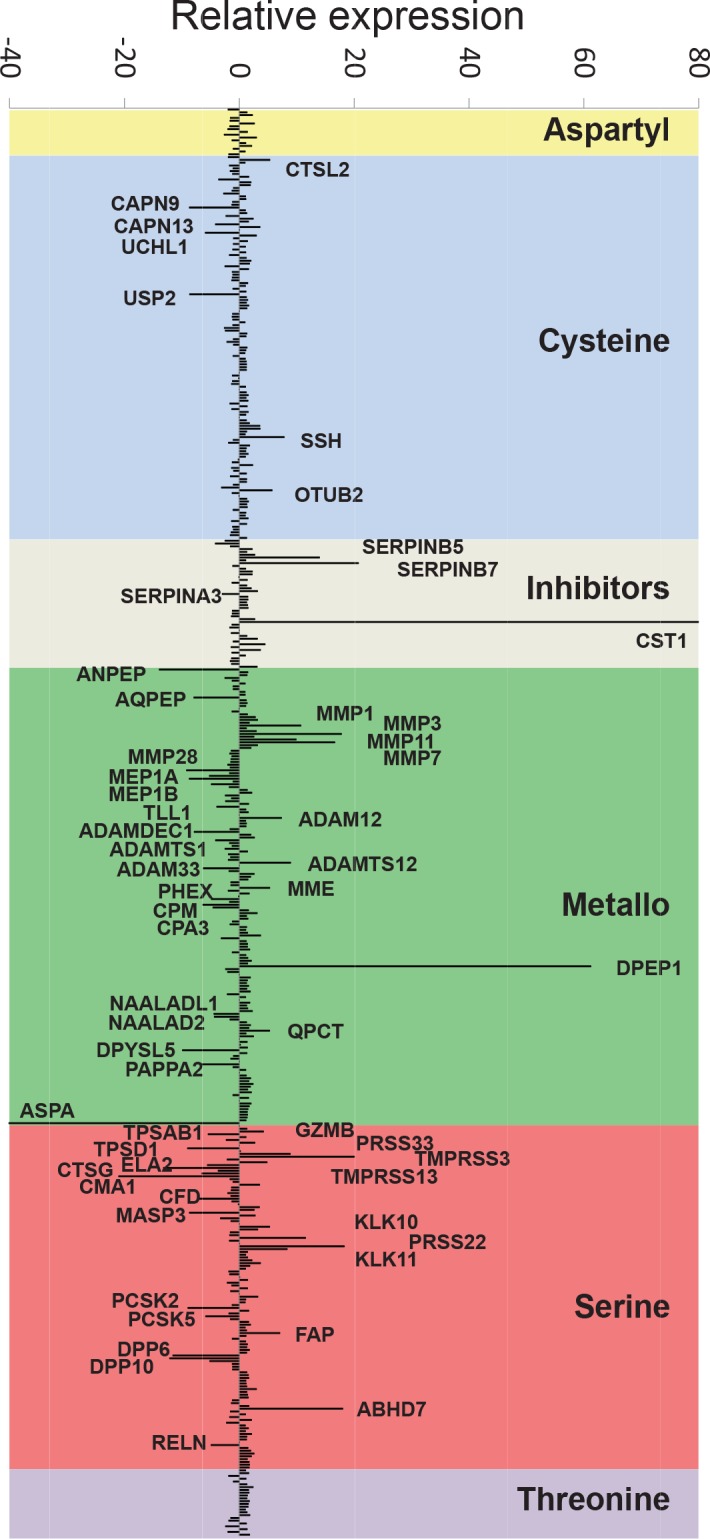
Degradome expression profiling of colorectal carcinoma Proteolytic genes are classified according to their catalytic class, and changes in the expression between colorectal carcinoma and normal mucosa are represented as median RQ (RQ=2^−ΔΔCt^) for genes upregulated in cancer samples and as −1/RQ for genes with higher expression in the normal tissue. Gene symbols are shown for those genes with RQ>4 or −1/RQ<−4.

To identify proteases differentially expressed in colon cancer, we obtained RNA from colon and rectal carcinomas as well as matched normal mucosa from 14 different patients diagnosed with colon cancer at different stages of progression, and subjected to surgery (Supplementary Table S1). Quantitative expression of human proteases and protease inhibitor genes was analyzed using two custom-designed TLDAs, with specific probes for 545 different human proteases, and 65 protease inhibitor genes. A comparison between tumor and normal samples resulted in the identification of genes with changes in expression of more than 4 RQs between tumor and normal samples. These included 21 protease genes overexpressed in tumor tissue, and 35 protease genes which were downregulated (Figure 1 and Table 1). Interestingly, we found a significant difference in the subcellular localization of proteases with altered expression in the tumor. This effect was evident for proteases overexpressed in tumor samples, as more than 90% of them have an extracellular localization (19 extracellular *vs.* 2 intracellular, p<0.001). This difference in the subcellular localization cannot be attributed to differences in the content of the arrays (266 *vs.* 264) nor to the normal expression of proteases in colon tissue, as intracellular and extracellular protease-coding genes are similarly expressed in these samples (219 extracellular *vs.* 255 intracellular). A similar trend was observed for protease genes downregulated in the tumor (24 extracellular *vs.* 11 intracellular, p=0.03). Additionally, analysis of protease inhibitors allowed us to identify three extracellular protease inhibitors overexpressed in tumor samples, while only one intracellular inhibitor was repressed (Table 1), suggesting that inhibitors follow a similar trend as proteases. Together, these data suggest that changes in the regulatory pathways in colon carcinoma cells predominantly affect proteases exerting their activity in the extracellular matrix and on the cell surface compartments.

Table 1Proteases and protease inhibitors differentially expressed in colorectal cancer

**Table:** Overexpressed in tumor tissue

Protease	Gene	RQ	Localization	Process	Activity
Dipeptidase 1 (renal)	DPEP1	61.2	EC-TM	Renal metabolism	Protease
Transmembrane protease, serine 3	TMPRSS3	20.0	EC-TM	Development of the inner ear	Protease
Brain serine proteinase 2	PRSS22	18.3	EC-S	Inmune response	Protease
Epoxide hydrolase 4	ABHD7	18.0	EC-TM	Unknown	NPH
Stromelysin 1	MMP3	17.8	EC-S	EC matrix degradation	Protease
Matrix metallopeptidase 7	MMP7	16.6	EC-S	EC matrix degradation	Protease
Kallikrein-related peptidase 10	KLK10	11.5	EC-S	EC matrix degradation	Protease
Matrix metallopeptidase 1	MMP1	10.7	EC-S	EC matrix degradation	Protease
Matrix metallopeptidase 11	MMP11	9.9	EC-S	EC matrix degradation	Protease
ADAMTS12	ADAMTS12	8.9	EC-S	EC matrix degradation	Protease
Protease, serine 33	PRSS33	8.9	EC-S	Unknown	Protease
Kallikrein-related peptidase 11	KLK11	8.3	EC-S	EC matrix degradation	Protease
Sonic hedgehog homolog (Drosophila)	SHH	7.8	EC-TM	Embryo patterning	Protease
ADAM metallopeptidase domain 12	ADAM12	7.3	EC-TM	Adipogenesis and myogenesis	Protease
Seprase	FAP	7.1	EC-TM	Inmune response	Protease
Otubain 2	OTUB2	5.7	IC	Unknown	Protease
Cathepsin L2	CTSL2	5.3	IC	Corneal physiology	Protease
Neprilysin	MME	5.3	EC-TM	Renal metabolism	Protease
Glutaminyl-peptide cyclotransferase	QPCT	5.3	EC-S	Peptides cyclization	NPH
Transmembrane protease, serine 13	TMPRSS13	4.8	EC-TM	Growth factor processing	Protease
Granzyme B	GZMB	4.2	EC-S	Inmune response	Protease
Cystatin SN	CST1	80.0	EC-S	Cysteine protease inhibitor	Inhibitor
serpin peptidase inhibitor, clade B, member 7	SERPINB7	20.7	EC-S	Serine protease inhibitor	Inhibitor
serpin peptidase inhibitor, clade B, member 5	SERPINB5	14.0	EC-S	Serine protease inhibitor	Inhibitor

**Table:** Downregulated in tumor tissue

Protease	Gene	RQ	Localization	Process	Activity
Aspartoacylase	ASPA	40.1	IC	NAA to aspartate and acetate conversion	NPH
Chymase 1, mast cell	CMA1	21.0	EC-S	EC matrix degradation	Protease
Alanyl (membrane) aminopeptidase	ANPEP	13.9	EC-TM	Digestion	Protease
Cathepsin G	CTSG	13.2	EC-S	Inmune response	Protease
Dipeptidyl-peptidase 10	DPP10	12.1	EC-TM	Voltage-gated potassium channels binding	NPH
Dipeptidyl-peptidase 6	DPP6	11.6	EC-TM	Voltage-gated potassium channels binding	NPH
Dihydropyrimidinase-related protein 5	DPYSL5	9.9	IC	Development. Differentiation	NPH
Matrix metallopeptidase 28	MMP28	9.2	EC-S	Tissue homeostasis. Wound repair	Protease
Tryptase delta 1	TPSD1	9.0	EC-S	Unknown	Protease
Proprotein convertase subtilisin/kexin type 2	PCSK2	8.9	IC	Prohormones processing	Protease
Meprin beta subunit	MEP1B	8.7	EC-TM	Unknown	Protease
Mannan-binding lectin serine peptidase 3	MASP3	8.7	EC-S	Lectin pathway of complement activation	Protease
Calpain 9	CAPN9	8.7	IC	Digestion	Protease
Ubiquitin specific peptidase 2	USP2	8.6	IC	Ubiquitin-dependent catabolic process	Protease
Aminopeptidase Q	AQPEP	7.9	EC-TM	Trophoblast implantation	Protease
ADAM-like, decysin 1	ADAMDEC1	7.9	EC-S	Dendritic cell function	Protease
Complement factor D (adipsin)	CFD	7.3	EC-S	Alternative complement pathway	Protease
Pappalysin 2	PAPPA2	6.8	EC-S	IGF processing	Protease
Granzyme M	GZMM	6.5	IC	Immune response	Protease
Carboxypeptidase M	CPM	6.4	EC-TM	Monocyte to macrophage differentiation	Protease
ADAM metallopeptidase domain 33	ADAM33	6.3	EC-TM	Asthma & bronchial hyperresponsiveness	Protease
Ubiquitin carboxyl-terminal esterase L1	UCHL1	5.9	IC	Ubiquitin-dependent catabolic process	Protease
Proprotein convertase subtilisin/kexin type 5	PCSK5	5.9	IC	Integrin processing	Protease
Elastase, neutrophil expressed	ELA2	5.6	EC-S	Immune response	Protease
Tryptase alpha/beta 1, tryptase beta 2	TPSAB1	5.4	EC-S	Immune response	Protease
Meprin alpha subunit	MEP1A	5.2	EC-TM	Unknown	Protease
Abhydrolase domain containing 12B	ABHD12B	5.1	IC	Esterase	NPH
Reelin	RELN	4.9	EC-S	Neural development	Protease
PHEX endopeptidase	PHEX	4.9	IC	Bone mineralization	Protease
Tolloid-like 1	TLL1	4.9	EC-S	Development. Differentiation	Protease
Carboxypeptidase A3	CPA3	4.6	EC-S	Secretory granule peptidase	Protease
N-acetylated α-linked acidic dipeptidase-like 1	NAALADL1	4.4	EC-TM	Neuropeptide alpha-NAAG hydrolysis	Protease
N-acetylated α-linked acidic dipeptidase 2	NAALAD2	4.4	EC-TM	Neuropeptide alpha-NAAG hydrolysis	Protease
Calpain 13	CAPN13	4.1	IC	Unknown	Protease
ADAM with thrombospondin type 1 motif, 1	ADAMTS1	4.1	EC-S	EC matrix degradation	Protease
Serpin peptidase inhibitor, clade A, member 3	SERPINA3	4.2	EC-S	Immune response	Inhibitor

IC, intracellular; EC, extracellular; S, secreted; TM, transmembrane; NPH, non-protease homologue

### Identification of novel proteases differentially expressed in colorectal cancer

The degradome expression profiling of colorectal cancer using TLDAs allowed us to identify several proteases with consistent changes in the expression pattern between tumor and normal tissue in different patients (Figure 1 and Table 1). As expected, among the overexpressed genes in tumor samples we found several genes previously identified as upregulated in this pathology, including several members of the matrix metalloproteinase family (*MMP1, MMP3, MMP7, MMP10, MMP11*), members of the kallikrein family of serine-peptidases (*KLK6, KLK8, KLK10*), as well as *DPEP1* or *PRSS22* among others [31-34]. The expression of some of these genes was up to 800 times higher in the tumor than in normal tissue, and their implication in colon cancer progression and invasion has already been demonstrated for some of them [35], validating the utility of degradome TLDAs to identify genes causally implicated in this pathology. Additionally, we were able to identify other genes highly upregulated in tumor samples, including *ABHD7, TMPRSS3* or *OTUB2*, which had not been previously associated with this pathology, and might constitute novel candidate genes for colon cancer. Similarly, a detailed analysis of genes downregulated in tumor samples resulted in the identification of protease genes including *MMP28, ANPEP, ADAMTS1* and *ADAMTS15*, previously known to be repressed in colon carcinoma or in other tumor types [36-38]. However, most of the genes with reduced expression in tumor samples had not been previously linked to this disease.

Although proteolytic enzymes have been widely characterized due to their pro-tumoral activities, recent reports have shown that some of these enzymes are tumor suppressors or have anti-tumoral effects [22, 24], suggesting that downregulation of their expression in tumor cells might contribute to tumor growth. In order to test this hypothesis, and to reduce the number of candidate genes, we first compared our list of the top fifty protease genes with altered expression in colon cancer to a list of genes somatically mutated in colon and breast cancers [27, 39]. This resulted in the identification of two upregulated genes (*DPEP1* and *MMP11*) as mutated in colon cancer and another three protease genes mutated in breast cancer (*TMPRSS3, ADAM12* and *MMP10*). In parallel studies for downregulated genes, we were able to identify seven genes with mutations in colon cancer (*DPP10, PCSK2, ADAM33, RELN, CAPN13, ADAMTS1* and *ADAMTS15*), and five genes with mutations in breast carcinomas (*MASP3, ABHD12B, TLL1, CPA3* and *DPP6*). The proportion of downregulated protease genes with somatic mutations in colon cancer was significantly higher when compared to upregulated ones (14% *vs.* 4%, p<0.05). This difference could not be attributed to changes in the transcript length between both groups (3396 bp vs. 4251 bp), suggesting that it might constitute a functional difference.

**Figure 2:**
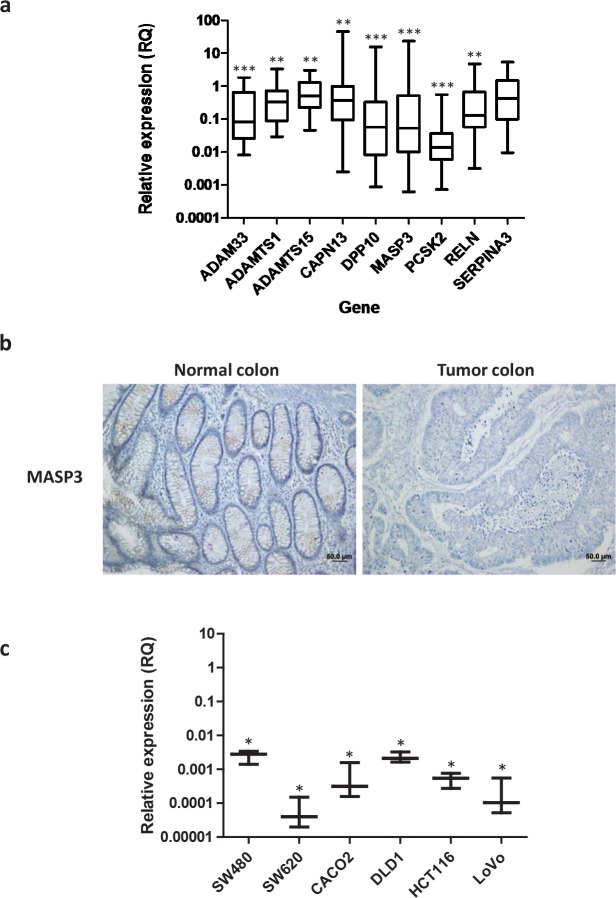
Identification of protease genes downregulated in colorectal cancer (a) Gene expression was quantified for nine different genes in a validation cohort of 28 colorectal carcinoma-normal mucosa pairs using qPCR. This led to the identification of proteolytic genes consistently downregulated in colorectal carcinomas. Fold change between tumor and normal tissue is expressed as RQ values and significant differences were assessed by a non-parametric Mann Whitney-Wilcoxon test (**, p<0.01; ***, p<0.001). (b) Immunohistochemical analysis of MASP3 expression in tumor and normal colorectal samples. MASP3 staining could be detected in normal colon mucosa (left panel), while no staining is detected in tumor tissue (right panel). (c) Expression of *MASP3* was quantified in different colorectal cancer cell lines. Colon fibroblasts were used as control for comparison. Data are expressed as fold change using RQ values. Significant differences were assessed by a non-parametric Mann Whitney-Wilcoxon test (*, p<0.05).

To try to confirm the repression of these genes in colorectal cancer, we performed qPCR analysis of this subset of genes in a validation series of 28 additional patients, consisting of pairs of tumor and matched normal mucosa (Supplementary Table S2). This analysis allowed us to corroborate that several of these protease-coding genes were expressed in normal colon mucosa, but consistently downregulated in colorectal carcinoma samples (Figure 2a). In fact, in some samples the expression of three of these genes, encoding the non-protease homologue DPP10, the proprotein convertase PCSK2 and the mannan-binding lectin serine peptidase 3 MASP3, are reduced up to a thousand fold in tumor tissue when compared to normal mucosa. No significant correlation between clinical stage and the expression of these genes could be established. Among the genes downregulated in colorectal carcinomas, we decided to focus on *MASP3*, as this gene encodes a *bona fide* protease whose participation in tumor processes has not been studied before, and its role in immunological processes could contribute to the tumorigenic process. To further confirm these results, we performed an immunohistochemical analysis of normal and tumor colorectal sections to investigate the expression of MASP3. Thus, in agreement with the qPCR results, MASP3 was absent in the tumor tissue while its expression could be detected in the corresponding normal colon mucosa (Figure 2b). Together, these results suggest that diminished activity of this protease, either through changes in gene expression or through the acquisition of somatic mutations, are common to colon tumors, and this gene could be causally implicated in the development of colorectal cancer.

### Antitumoral effects of MASP3 in colorectal carcinomas

**Figure 3:**
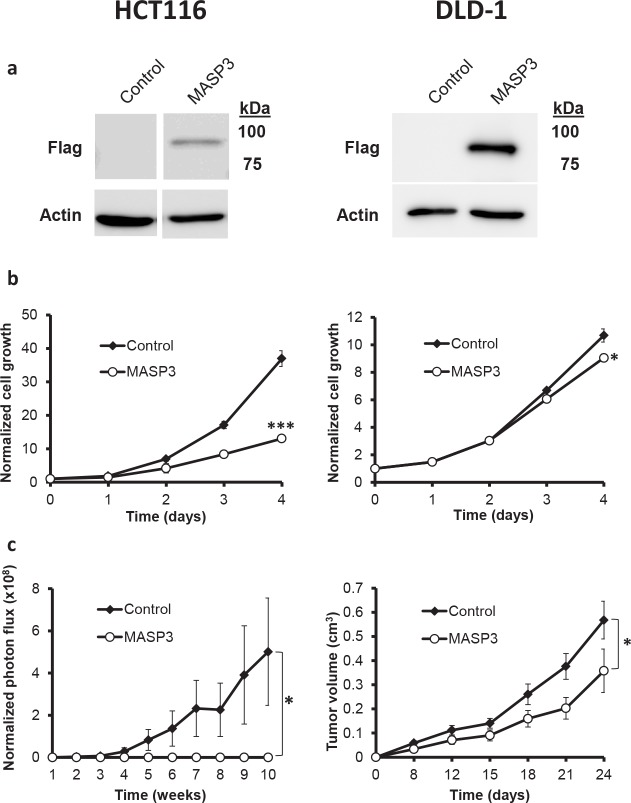
MASP3 expression reduces the proliferation of colorectal cancer cells and restrain tumor growth in a xenograft mouse model. HCT116 Luc2 (left side) and DLD- 1 (right side) colorectal cancer cells were transfected with a vector expressing *MASP3* cDNA, or with the empty vector as control. (a) Western-blot analysis showing the overexpression of *MASP3* in transfected cells. (b) Cell proliferation was quantified using an MTT-based assay. Each point was normalized with respect to 0 h and the mean ± SEM was represented (*, p<0.05; ***, p<0.001). Expression of *MASP3* resulted in a reduction in cell proliferation when compared to control cells. (c) Tumor xenograft experiments were carried out with cells overexpressing *MASP3* or transfected with the empty vector. Normalized photon flux ± SEM (HCT116 Luc2) or tumor volume ± SEM (DLD-1) was calculated for each group at the indicated times after injection and significant differences were assessed by a linear mixed-effects model (*, p<0.05). Ectopic expression of *MASP3* blocked tumor formation compared to cells transfected with empty vector.

To further characterize this hypothesis as well as to perform functional analysis of this gene *in vitro* and *in vivo*, we studied the expression of *MASP3* in different colorectal cancer cell lines (CaCo2, DLD-1, HCT116, LoVo, SW480, SW620), as well as in colon fibroblasts. This analysis revealed that *MASP3* is expressed in normal colon fibroblasts, while its expression is significantly reduced in all six cancer cell lines examined, reaching changes greater than 1,000-fold (Figure 2c). These results suggest that these cell lines represent an adequate model to investigate whether expression of this protease might repress tumor growth *in vitro* or *in vivo*. To do that, we transfected the colon carcinoma cell lines HCT116 Luc2 and DLD-1 with an expression vector encoding *MASP3*, as well as with the empty vector. The overexpression of this gene was confirmed by Western Blot analysis (Figure 3a).

Overexpression of *MASP3* significantly reduced cell proliferation *in vitro* when compared to control cells (Figure 3b). In order to investigate if the observed reduction in proliferative potential was actually due to possible pro-apoptotic effects of this protein, we decided to compare the levels of apoptosis between HCT116 cells overexpressing *MASP3* and control cells. The analysis of PARP1 caspase cleavage products revealed no differences between both types of cells (Supplementary Figure S1), indicating that the antiproliferative action of *MASP3* is not derived from pro-apoptotic effects. Next, to investigate the *in vivo* significance of these results, we assayed the impact exerted by the ectopic expression of this protease on the tumorigenic potential of colon cancer cells, using a mouse xenograft model. Thus, HCT116 Luc2 cells transfected with the empty vector readily yielded fast-growing tumors when injected subcutaneously in nude mice. By contrast, overexpression of *MASP3* in this cell line abrogated its ability to produce detectable tumors in the same experiments (Figure 3c). *MASP3*-overexpressing DLD-1 cells produced detectable tumors, but their growth was significantly reduced when compared to control cells (Figure 3c).

**Figure 4:**
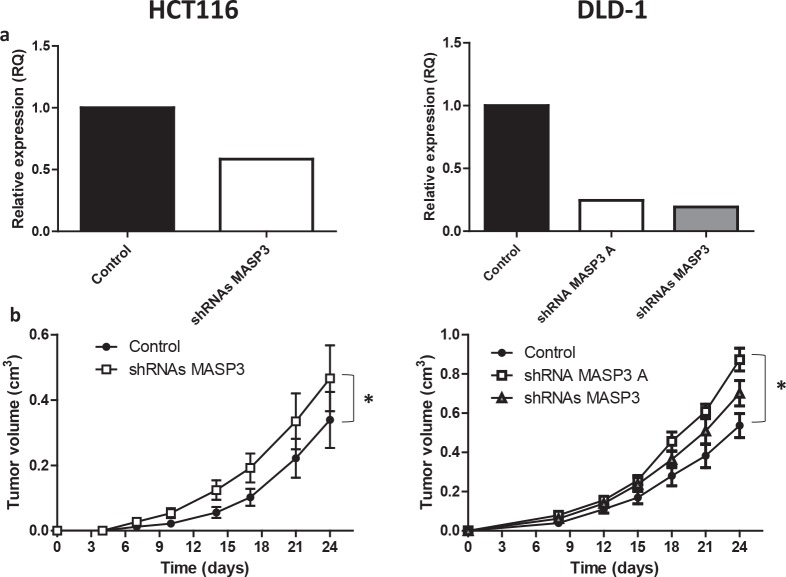
MASP3 downregulation increases the tumorigenic potential of colorectal cancer cells. HCT116 (left side) and DLD-1 (right side) colorectal cancer cells were transduced with *MASP3* shRNA vectors or with the empty vector as control. (a) The relative expression levels of *MASP3* were assayed by quantitative RT-PCR. (b) Tumor xenograft experiments were carried out with *MASP3*-silenced and control cells. Tumor volume was calculated for each group at the indicated times after injection and significant differences were assessed by a linear mixed-effects model (*, p<0.05). shRNAs MASP3 denotes the pool of 4 available silencing vectors, shRNA MASP3 A denotes the most efficient individual vector.

As a complementary approach and due to the identification of *MASP3* as a gene consistently downregulated in colon carcinomas, we investigated whether depletion of this gene could increase the tumorigenic potential of HCT116 and DLD-1 cancer cells. Silencing of *MASP3* by transduction with lentiviral vectors encoding specific shRNAs resulted in a significant increase in tumor growth rate when compared to control cells (Figure 4). Together, these data suggest that *MASP3* downregulation in colon carcinomas might be a required step in the development of the malignant phenotype by colon cancer cells, facilitating cell proliferation and tumor growth.

## DISCUSSION

Proteases play critical roles in cancer biology, and all steps of cancer progression, from cell transformation to tumor invasion and metastasis, depend on proteolytic activities [8-10]. In consequence, alterations in sequence or expression of protease-encoding genes are probably a nearly universal feature of cancer, prompting us to hypothesize that most, if not all, human cancers present “protease addiction”, i.e. an exacerbated dependence on the activity of specific proteolytic enzymes, which potentially constitute valuable targets for anti-cancer therapies [40]. Importantly, not all proteases relevant to cancer biology play cancer-promoting roles, but a growing number of them present anti-tumor properties [22-26]. Taken together, these facts support the interest of implementing reliable procedures for profiling protease expression in human cancers.

Different approaches are currently available that allow global transcriptional profiling of biological samples. Most studies performed in this direction have been based on the use of microarray hybridization platforms, which provide a nearly complete coverage of the human genome, including the degradome. Over the last 15 years, such studies have represented the most powerful tool for global analysis of gene expression and they have generated expression data from tens of thousands of tumor samples [41]. The recent development of RNA-Seq has provided an alternative approach for global transcriptional profiling, with the clear advantage of allowing to detect the expression of previously uncharacterized loci [42]. In this work we have developed a quantitative PCR-based approach for profiling the entire degradome, with the advantages of simplicity, sensitivity and reduced cost compared to the mentioned global approaches.

TLDA-based degradome profiling of colorectal carcinomas uncovered significant transcriptional changes, which preferentially affected to extracellular proteases and inhibitors. This enrichment of extracellular molecules among both overexpressed and downregulated transcripts points to the relevance of the extracellular medium in cancer biology and highlights the relevance of the degradome in its regulation. Remarkably, a significant proportion of protease genes found in this work to be downregulated in colorectal cancer had been reported to be somatically mutated in colorectal or breast cancer. Among these genes, we chose to focus on *MASP3*, which encodes a *bona fide* protease, whose immunological role could be involved in tumor progression. Overexpression of this enzyme in two different colorectal cancer cell lines led to a marked drop of their proliferation in cell culture and to the reduction or abrogation of their tumorigenicity when xenografted in nude mice. Remarkably, shRNA-mediated silencing of *MASP3* increased the tumorigenicity of the assayed colorectal cancer cell lines, despite the already very low basal expression level in these cells. These results, along with the consistently reduced expression of this protease in colorectal carcinomas and cancer cell lines, demonstrate its anticancer effects. It is noteworthy that the experimental system used in this work allowed us to investigate the biological relevance of MASP3 produced exclusively by the cancer cells, regardless of the levels of this protease synthesized by stromal or inflammatory components of the tumor. The above results indicate that the antitumorigenic effect of MASP3 does not require an alteration of its levels in stromal or inflammatory cells, since these components are expected to be the same in animals injected with control cells or with protease-overexpressing cells.

To date, very little is known about the role of MASP3 in tumor development. MASP3 is a mannan-binding lectin-associated serine protease with an immunological role through activation of the complement system [43]. Three different human MASP proteins have been described. MASP1 and 3 are encoded by the same gene, generated by alternative splicing [43]. They share the same heavy chain, but have a different light chain. Even though the expression of both isoforms has been quantified in our TLDA-based study, only *MASP3* showed significant changes in tumor samples compared to normal mucosa. Based on the role of MASP proteins in complement activation, we hypothesized that complement could be involved in MASP3 antitumoral effects. As a first approach to investigate this possibility, we tested in parallel the effects of MASP3 overexpression and silencing on colon cancer proliferation using heat inactivated and non-inactivated serum in the culture medium. Since we did not observe any significant difference in these experiments, we cannot conclude that complement is involved in this effect. Natural substrates have not been identified for MASP3, but it has been reported to cleave recombinant insulin-like growth factor binding protein 5 (IGFBP-5) *in vitro* [44]. IGFBP-5, through its binding to IGFs and the regulation of their interaction with IGF receptors, is an essential regulator of several physiological processes [45-46]. Thus, MASP3-mediated cleavage of IGFBP-5 might hinder its ability to bind IGF-I suggesting that this linkage between MASP3 and IGFs could constitute an important mechanism for colon cancer progression. We have explored this possibility by studying the maturation of IGFBP-5 in cells overexpressing *MASP3*, finding that the IGFBP-5 processed products and their relative levels were indistinguishable in control and *MASP3*-overexpressing cells (data not shown), indicating that processing of this IGF binding protein does not account for the antitumorigenic effects of MASP3 in colorectal cells. The lack of information on the biochemical properties and function of this protease prevents the design of hypothesis-driven experiments to explore the mechanisms underlying its antitumoral effects in colorectal cells and further studies will be required to identify the MASP-3 substrates involved in its anticancer effects.

In summary, in this work we have designed a new qPCR-based tool for profiling the human degradome in normal and pathological conditions, including cancer. We have used these TLDAs to evaluate protease expression levels in colorectal carcinomas with the finding that most proteases with altered expression in these tumors exert their function in the extracellular compartment. In addition, we have found that among repressed proteases there was a higher proportion of genes with somatic mutations in colorectal cancer when compared to upregulated proteases. Further studies with some of these genes have demonstrated that they are consistently repressed in colorectal carcinomas and cancer cell lines when compared to normal colonic mucosa. Additionally, functional analysis have confirmed that *MASP3* is a *bona fide* candidate antitumor protease, as it is either mutated or downregulated in tumor samples, and it plays a role in the regulation of cell proliferation and subcutaneous tumor formation. Taken collectively, these findings strongly support the utility of degradome profiling to identify novel proteases with antitumor functions and provide new insights into the functional diversity of proteases associated with human malignancies.

## METHODS

### Low-density array design

The human degradome database [47] was used to design two TaqMan Low Density Arrays (TLDA) containing specific primers and probes for all human proteases and a subset of protease inhibitors (Applied Biosystems). An EP-array was designed for the expression profiling of extracellular proteases and inhibitors, and membrane-bound proteases. Additionally, we designed an IP-array for the analysis of intracellular proteases and inhibitors. As a quality control to measure the number of probes able to detect the expression of a human protease, we performed qPCR with these arrays and using as starting material cDNAs derived from 10 different human tissues (brain, colon, liver, lung, muscle, pancreas, peripheral blood leukocytes, prostate, stomach and testis). Using this approach, more than 96.5% (608/630) of the probes were able to detect the expression of a protease gene in at least some of these tissues.

### RNA samples and qPCR amplification

Colon and rectal carcinoma samples, as well as matched normal mucosa from the same patients were obtained from the Tumor Bank of the Hospital Universitario Central de Asturias (Oviedo, Spain) and from Hospital Clinic (Barcelona, Spain). Surgical specimens were collected in the operating theatre immediately after their removal, and they were quickly transported in an unfixed state to the pathology department. Samples in which time to freezing exceeded 30 min were discarded. Areas with massive ischaemic and/or necrotic phenomena were avoided. Samples were embedded in OCT medium (Bayer), and snap-frozen on liquid nitrogen-freezing isopentane. RNA was obtained from 10 mg of frozen tissue using an RNA purification kit from Qiagen, and the purity and integrity of the RNA was determined using a BioAnalyzer (Agilent Technologies). Only RNA samples with a RIN>=7 were used for degradome expression profiling. One μg of total RNA was used for cDNA synthesis, and qPCR amplification was performed following manufacturer's instructions (Applied Biosystems), using an Applied Biosystems 7900HT Fast Real-Time PCR system. Data analysis was performed with SDS 2.1 (Applied Biosystems).

### Immunostaining

Normal and tumor colorectal samples (n=40) were obtained from the Tumor Bank of the Hospital Universitario Central de Asturias (Oviedo, Spain) and used to evaluate the expression of the most downregulated genes (*MASP3, DPP10* and *PCSK2*). Pilot experiments revealed that MASP3 protein expression was reproducibly detected in normal mucosa samples, while antibodies against DPP10 and PCSK2 failed to yield a reliable signal in these samples. Therefore, we decided to focus our analysis on MASP3. The automated system DISCOVERY® (Ventana Medical System, Tucson, AZ) was used to carry out the immunohistochemical protein detection. Sections were deparaffinized and rehydrated in EZ Prep® (Ventana Medical System) for 20 minutes. Antigen retrieval was done by heating (CC1 HCl-Tris buffer solution, pH 9.0) (Ventana Medical System). Endogenous peroxidase activity was blocked with H_2_O_2_ solution (Inhibitor^®^, Ventana Medical System) for 4 minutes. Samples were incubated with primary antibody at 37 °C with polyclonal anti-MASP3 (Atlas Antibodies). Slides were incubated with the secondary antibody (OmniMap^®^ Ventana medical System) for 30 minutes at room temperature. Then, samples were visualized with DAB (3-3'-Diaminobenzidine) (Ventana Medical System). Finally, samples were counterstained with hematoxyline (Ventana Medical System), dehydrated and mounted in Entellan^®^ (Merck, Germany). Sections were studied and photographed (20X) under a light microscope (Nikon-Eclipse 80i).

### DNA constructs

Vector containing human cDNA from *MASP3* was purchased from GeneService. The cDNA was tagged with two consecutive FLAG epitopes in the carboxy terminus and subcloned into the pcDNA3.1 and pCEP-Pu expression vectors. The constructs were verified by capillary sequencing. For RNA interference experiments, four shRNA vectors were purchased for *MASP3* (Open Biosystems), and their ability to repress the expression of the protease gene was evaluated by quantitative RT-PCR on RNA samples from cells transduced with the respective vectors, individually and combined. Due to the very low basal expression of this protease, only the complete set combined or the vector designed as *shRNA MASP3 A* produced a significant silencing of this protease in colon cancer cells.

### Cell culture

Tumor cell lines 293T, HCT116, SW480, SW620, LoVo, DLD-1, and Caco-2 were purchased from the American Type Culture Collection. The luciferase-expressing cell line HCT116 Luc2 was purchased from Caliper Life Sciences. Cells were routinely maintained in Dulbecco's modified Eagle's medium (DMEM) containing 10% heat-inactivated fetal bovine serum (FBS), 100 U/ml penicillin and 50 μg/ml streptomycin.

### Transfection

HCT116 Luc2 and DLD-1 cells were transfected with a *MASP3* cDNA cloned into pcDNA3.1 and pCEP-Pu, respectively, using Lipofectamine reagent (Invitrogen). Cells were grown at 37 °C for 2 days in culture medium before changing to selection medium, 1 mg/ml G418 (Invitrogen) for HCT116 Luc2 and 1 μg/ml Puromycin (Sigma) for DLD-1 cells, respectively. After selection, cell populations expressing the desired gene were obtained along with control cell lines transfected with the empty vectors.

### Viral package and cell infection

Lentiviruses were packaged in HEK-293T cells using a VSVG-based package system kindly provided by Dr JM Silva (Columbia University, New York, USA). Cells were transfected using TransITs-LT1 Transfection Reagent (Mirus) and a mixture of 2 μg of the desired plasmid and 1 μg of each lentiviral helper, following the manufacturer's instructions. Transfection medium was removed 24 h after transfection and fresh medium was added to the plate. Cell supernatants were collected at 24 and 48 h and filtered through a 0.45-μm sterile filter. HCT116 and DLD-1 cells were seeded in 6-well plates at 20–30% confluence 24 h before infection. The following day, 1 ml of viral supernatant supplemented with 5 mg/ml of polybrene (Millipore) was added to growing cells. This step was repeated twice and cells were left recovering for 24 h in growing media before puromycin selection (1 μg/ml).

### Western Blot analysis

Forty eight hours after transfection, cells were washed twice with 1X PBS and lysed in 50 mM Tris buffer, pH 7.4, containing 150 mM NaCl, 1% Triton X-100, 10 mM EDTA, and complete protease inhibitor cocktail (Roche Applied Science). The protein concentration was evaluated by the bicinchoninic acid technique (BCA protein assay kit; Pierce Biotechnology Inc.). A protein sample (10 μg) was loaded on SDS-polyacrylamide gels. After electrophoresis, gels were electrotransferred onto PVDF filters, and the filters were blocked with 5% nonfat dried milk in TBS-T (TBS with 0.05% Tween-20) and incubated with an anti-FLAG antibody following the recommendations of the supplier (Cell Signalling). After 3 washes with TBS-T, filters were incubated with the corresponding secondary antibody in 1.5% nonfat dry milk in TBS-T, and developed with Immobilon Western Chemiluminescent HRP substrate (Millipore) in a LAS-3000 Imaging System (Fujifilm).

### Proliferation assay

To quantify cell proliferation, a Cell Titer 96 Non Radioactive cell proliferation kit was used following manufacturer's instructions (Promega Corp.). Briefly, HCT116 Luc2 and DLD-1 transfected cells were seeded into 96-well plates at a density of 5×10^3^ cells per well (100 μl) and incubated at 37 °C, 5% CO_2_ for 4 days. Cell proliferation was quantified by measuring the conversion of a tetrazolium salt into formazan in living cells. At the desired time points (0 h, 24 h, 48 h and 72 h), 15 μl of Dye solution was added into each well (n=4) and cells were incubated at 37 °C for 2 h. Then, 100 μl of solubilization/stop mixture was added into each well. After 1 h of incubation at 37 °C the absorbance was measured at 570 nm with a Power Wave XS Microplate reader (Biotek). Then, each point was normalized with time 0 h and mean ± SEM was calculated and represented. Statistical significance was assessed using a non-parametric Mann Whitney-Wilcoxon test (*, p<0.05; ***, p<0.001).

### Mouse xenograft model

Six-week-old athymic Nude-Foxn1^nu/nu^ mice (Charles River) were used for tumor xenograft experiments with HCT116 Luc2 and DLD-1 cell lines, transfected with pcDNA3 or pCEP-Pu constructs or HCT116 and DLD-1 cell lines transduced with shRNA vectors. Eight mice were used for each experiment. Both flanks of each animal were injected subcutaneously with 2 × 10^6^ (HCT116 Luc2) or 3 × 10^6^ (DLD-1) cells in 100 μl PBS resulting in eight flanks per construction. To monitor growth of tumors derived from HCT116 Luc2 cells (transfected with the *MASP3* expression vector or with empty pcDNA3.1), tumor size was measured every week by chemiluminescence detection using a Xenogen IVIS system (Caliper Life Sciences). Normalized photon flux ± SEM was calculated for each group. For xenografts of colon cancer cells without a luciferase reporter, tumor size was measured twice a week with a caliper and tumor volume was determined using the formula: V=0.4 × A × B^2^, where A is the largest and B is the smallest dimension of the tumor. For both types of measurements, significant differences were assessed by a linear mixed-effects model (*, p<0.05).

## Supplementary Tables and Figure


