# Supplementary material for: Identification of novel tumor suppressor proteases by degradome profiling of colorectal carcinomas
Source: Oncotarget. 2013 Sep 15;4(11):1919–32. doi: 10.18632/oncotarget.1303 (PMC3875759; doi:10.18632/oncotarget.1303)
Supplement: Supplementary file 1 [file oncotarget-04-1919-s001.pdf]

Supplementary Table 1. Clinicopathologic features of the discovery series (N=14)		
	N	(%)
**Age (Mean=71; Min=21; Max=91)**		
<Mean	6	43
>Mean	8	57
**Gender**		
Male	9	64
Female	5	36
**Tumor Size (Mean=5 cm)**		
< Mean	9	64
> Mean	5	36
**Vascular Invasion**		
Absent	11	79
Present	3	21
**Perineural Invasion**		
Absent	11	79
Present	3	21
**Stage (AJCC/UICC)**		
I	3	21
II	7	50
III	4	29
IV	0	0
**Histological Differentation**		
Well	2	14
Moderate	11	79
Poor	1	7
**Tumor Localization**		
Ascending Colon	1	7
Transverse Colon	1	7
Descending Colon	2	14
Sigmoid Colon	5	36
Cecum	3	21
Rectum	2	14

Supplementary Table 2. Clinicopathologic features of the validation series (N=28)	N	(%)
**Age (Mean=71; Min=54; Max=91)**		
<Mean	14	50
>Mean	14	50
**Gender**		
Male	19	68
Female	9	32
**Tumor Size (Mean=5.8 cm)**		
< Mean	14	50
> Mean	14	50
**Vascular Invasion**		
Absent	21	75
Present	7	25
**Perineural Invasion**		
Absent	26	93
Present	2	7
**Stage (AJCC/UICC)**		
I	3	11
II	12	43
III	10	35
IV	3	11
**Histological Differentation**		
Well	2	7
Moderate	15	54
Poor	11	39
**Tumor Localization**		
Ascending Colon	2	7
Transverse Colon	1	4
Descending Colon	2	7
Sigmoid Colon	8	29
Cecum	10	35
Rectum	5	18

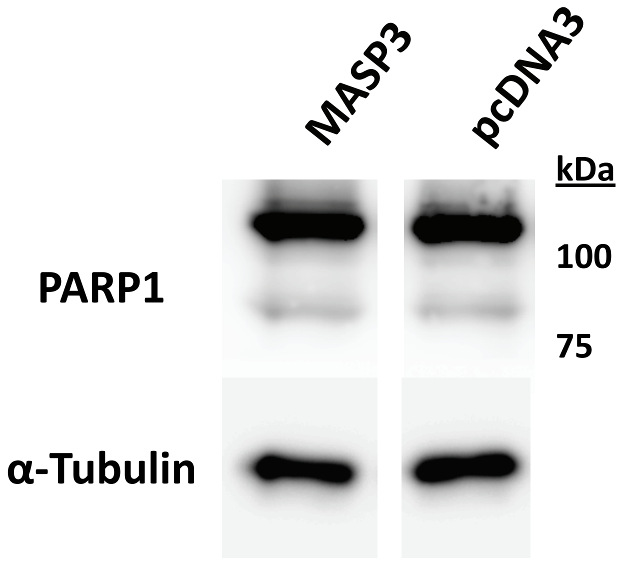

Supplementary Figure S1. *MASP3* overexpression does not increase apoptosis in HCT116 colon cancer cells. To assess apoptosis levels in HCT116 cells transfected with *MASP3* or control vector, the large fragment (89 kDa) of PARP1 protein produced by caspase cleavage was analyzed by Western Blot with an anti-PARP antibody (Cell Signaling). An anti-α-Tubulin antibody (Sigma-Aldrich) was used as a loading control.
